# The FSGS protein actinin-4 interacts with NKCC2 to regulate thick ascending limb NaCl reabsorption

**DOI:** 10.1152/ajprenal.00119.2024

**Published:** 2024-10-24

**Authors:** Dipak Maskey, Tang-Dong Liao, D’Anna L. Potter, Pablo A. Ortiz

**Affiliations:** ^1^Hypertension and Vascular Research Division, Department of Internal Medicine, Henry Ford Hospital, Detroit, Michigan, United States; ^2^Department of Physiology, Integrative Bioscience Center, Wayne State University, Detroit, Michigan, United States

**Keywords:** FSGS protein actinin-4, NaCl reabsorption, NKCC2 trafficking, protein-protein interaction, thick ascending limb

## Abstract

In the kidney, the thick ascending limb (TAL) of the loop of Henle plays a vital role in NaCl homeostasis and blood pressure regulation. In human and animal models of salt-sensitive hypertension, NaCl reabsorption via the apical Na^+^/K^+^/2Cl^−^ cotransporter (NKCC2) is abnormally increased in the TAL. We showed that NaCl reabsorption is controlled by the presence of NKCC2 at the apical surface of TALs. However, the molecular mechanisms that maintain the steady-state levels of NKCC2 at the apical surface are not clearly understood. Here, we report that NKCC2 interacts with the F-actin cross-linking protein actinin-4 (ACTN4). We find that ACTN4 is expressed in TALs by Western blot and immunofluorescence microscopy. ACTN4 immunoprecipitated with NKCC2 and recombinant glutathione-*S*-transferase (GST)-ACTN4 pulled down NKCC2 from TAL lysates. ACTN4 is involved in endocytosis in other cells. Therefore, we hypothesized that ACTN4 binds apical NKCC2 and regulates its trafficking. To study the role of ACTN4 in NKCC2 surface expression, we silenced ACTN4 in vivo via shRNA or CRISPR/Cas9 system to decrease ACTN4 expression in TALs. We observed that silencing ACTN4 in vivo via shRNA or CRISPR/Cas9 system increased the amount of NKCC2 at the apical surface of TALs. Consistent with an increase in surface NKCC2, bumetanide-induced diuresis and natriuresis were enhanced by 35% after silencing of ACTN4 in vivo (AV-NKCC2-Cas9: 3,841 ± 709 vs. AAV-gRNA-ACTN4: 5,546 ± 622 µmol Na/8 h, *n* = 5, *P* < 0.05). We conclude that ACTN4 binds NKCC2 to regulate its surface expression. Selective depletion of ACTN4 in TALs using shRNA or CRISPR/Cas9 enhances surface NKCC2 and TAL-NaCl reabsorption, indicating that regulation of the ACTN4-NKCC2 interaction is important for renal NaCl reabsorption and could be related to hypertension.

**NEW & NOTEWORTHY** ACTN4 function and dysfunction in glomerular podocytes have been extensively studied. However, the function of ACTN4 in the nephron has not been studied. Our paper shows for the first time that ACTN4, in the nephron, regulates NaCl reabsorption in part by affecting NKCC2 surface expression. Protein-protein interactions between ACTN4 and NKCC2 seem to mediate NKCC2 endocytosis in TALs. When ACTN4 was silenced in the TAL in vivo using CRISPR/Cas9 or shRNAs, surface NKCC2 and NaCl reabsorption increased.

## INTRODUCTION

Renal cells of the thick ascending limb (TAL) of the loop of Henle (1) reabsorb up to 25%–30% of the filtrated NaCl of urine via apical Na^+^/K^+^/2Cl^–^ cotransporter (NKCC2) to maintain salt and fluid homeostasis (2). Under baseline conditions, NKCC2 is present at the apical surface and subapical space of TAL, including macula densa cells of the kidney (1, 3, 4). There is a tight relationship between the presence of active cotransporter NKCC2 at the apical membrane in TAL and the capacity of the TAL to reabsorb NaCl (5–7). Our laboratory demonstrated that the presence of NKCC2 in the apical membrane and NaCl transport are tightly correlated (5, 8–10) and the levels of NKCC2 in the apical membrane are regulated by constitutive NKCC2 endocytosis (9). Evidence shows that a fraction of the NKCC2 retrieved pool is recycled back to the plasma membrane of apical cells after NKCC2 internalization (9). Disruption of NKCC2 endocytosis induces accumulation of NKCC2 expression at the cell membrane, which increases NKCC2 activity and enhances the NaCl reabsorption in TAL (9), underlining the importance of NKCC2 endocytosis in NaCl homeostasis (11). However, the molecular mechanisms that mediate enhanced NKCC2 activity in TALs are poorly understood.

Our laboratory previously showed that a small percentage (3%–5%) of NKCC2 is at the apical membrane of the TAL. Expression of surface NKCC2 is critical for NaCl reabsorption in the TAL and tightly regulated via trafficking into and out of the apical membrane (5, 11). The cellular and molecular mechanisms that keep NKCC2 in a steady state at the apical membrane in TAL depend on the combinatorial actions of exocytosis (10), endocytosis, and recycling (9, 12). Any gene or protein affecting these processes could potentially increase the expression of NKCC2 at the apical surface of TAL and enhance its activity. Our previous studies indicated that VAMP2 and VAMP3 are involved in exocytosis (13), whereas Alstrom syndrome 1 protein (ALMS1) (14), dynamin 2, and caveolin are involved in endocytosis (15).

We previously found that NKCC2 interacts with ALMS1 in the TAL (14). Genetic deletion of ALMS1 in rats decreased endocytosis and enhanced surface expression of NKCC2 and TAL-mediated NaCl reabsorption, which increased blood pressure and enhanced salt sensitivity. Pull-down of ALMS1 with TAL lysates followed by LC-mass spectrometry identified actinin-4 (ACTN4), a protein primarily involved in podocyte function, as an interacting partner. Genetic deletion of ACTN4 is known to cause glomerular damage (16), but its function in nephron salt handling is unclear. Thus, it is conceivable that ACTN4 could mediate NKCC2 trafficking by interacting with it. There have been no direct studies examining the role of ACTN4 on TALs. However, in podocytes (17), mutations in ACTN4 cause a highly penetrant autosomal dominant familial focal and segmental glomerulosclerosis (FSGS) (18), which is associated with chronic kidney disease and kidney failure (19). In other cells, ACTN4 mediates endocytosis of other proteins through direct binding to endocytic adaptor proteins (20) or through the Arp2/3 complex (21). Therefore, we hypothesized that ACTN4 binds with NKCC2 and other trafficking proteins in the TAL, and regulates NKCC2 surface expression and TAL-NaCl reabsorption. To test this hypothesis, we performed a targeted proteomic screening for ACTN4-binding proteins from freshly isolated medullary TALs and identified NKCC2 as an interacting partner of ACTN4. We report for the first time that silencing of ACTN4 in vivo enhances surface expression of NKCC2 and TAL-mediated NaCl reabsorption.

## MATERIALS AND METHODS

### Reagents and Antibodies

Reagents for steady-state surface biotinylation were obtained from ThermoFisher Scientific Inc. (Waltham, MA), and reagents for glutathione-*S*-transferase (GST) pull-down were purchased from GE Life Sciences. GST-ACTN4 plasmids were gifted by Dr. Hung-Ying Kao (Department of Biochemistry, Case Western Reserve University). The rabbit anti-C-terminus NKCC2 antibody (1:10,000) was produced by our laboratory against a sequence unique to NKCC2 (residues 859–873 rat NKCC2) and characterized by our laboratory (9, 12) and others (22). The polyclonal rabbit anti-ACTN4 antibody (1:1,000) was gifted by Dr. Vivian Tang (23), and the ACTN4 monoclonal antibody (1:50) (clone D3) was purchased from Santa Cruz Biotechnology (Dallas, TX). The rabbit Alexa-Fluor 488-conjugated (1:200) and anti-mouse Alexa-Fluor 568-conjugated antibodies (1:200) were obtained from Invitrogen. The horseradish peroxidase (HRP)-conjugated secondary anti-mouse and anti-rabbit antibodies (1:2,000) were obtained from GE Healthcare, and rabbit nonimmune IgG (1:200) was from Sigma.

### Suspension of Medullary TALs

We prepared the suspension of medullary TALs as reported previously. Briefly, we received 70–100 g male Sprague-Dawley rats (Charles River Laboratories). Animals were fed a standard diet (0.22% Na^+^, 1% K^+^) from Envigo. Medullary TAL suspensions were prepared according to previously described methods (8). Animals were anesthetized with ketamine (100 mg/kg body wt ip) and xylazine (20 mg/kg body wt ip). Briefly, the abdominal cavity was opened, and the kidneys were perfused retrograde via the aorta with a physiological solution containing 0.1% collagenase (Sigma, St. Louis, MO) and 100 U/mL heparin. The inner stripe of the outer medulla was cut from coronal slices, minced, and incubated at 37°C for 30 min with 0.1% collagenase in a physiological saline solution and gassed every 5 min with 100% O_2_. Tissue was pelleted by gentle centrifugation at 150 *g* for 2 min, resuspended in chilled physiological solution, and stirred on ice for 30 min to release the tubules. The suspension was filtered through a 250-μm nylon mesh and centrifuged at 150 *g* for 2 min. The pellet was washed, centrifuged again, and finally resuspended in 0.4 mL chilled physiological solution. The composition of the physiological solution was 130 mM NaCl, 2.5 mM NaH_2_PO_4_, 4.0 mM KCl, 1.2 mM MgSO_4_, 5 mM l-alanine, 1.0 mM Na-citrate, 5.5 mM glucose, 2.0 mM Ca-lactate, and 10 mM HEPES, pH 7.40.

All protocols were approved and conducted in accordance with Institutional Animal Care and Use Committee guidelines (IACUC) of Wayne State University.

### Steady-State Surface NKCC2 Levels

Cell surface biotinylation of mTAL suspensions was performed as previously described (12–14, 24–27). Briefly, tubule suspensions were equilibrated for 20 min at 37°C and gassed every 5 min with 100% O_2_. After equilibration, TALs were incubated with 0.75 mL chilled biotinylation solution (HEPES-Ca^2+^-Mg^2+^ buffer: 10 mM HEPES, 130 mM NaCl, 2 mM MgSO_4_, 1 mM CaCl_2_, 5.5 mM glucose, pH 7.8–8.0) containing 0.9 mg/mL NHS-SS-biotin (Pierce Biotechnology) in a rocker platform at 4°C for 15 min. Then, 0.75 mL of freshly prepared NHS-SS-biotin (0.9 mg/mL) was added on top, and the samples were incubated for an additional 15-min period. After biotinylation, tubules were washed three times at 4°C with a physiological solution containing 100 mM glycine to remove the excess NHS-SS-biotin. TALs were centrifuged (150 *g*) and lysed in buffer containing 150 mM NaCl, 50 mM HEPES, and 5 mM EDTA, plus 2% Triton X-100 and 0.1% SDS, pH 7.5, and protease inhibitors [10 μg/mL aprotinin, 5 μg/mL leupeptin, 4 mmol/L benzamidine, 5 μg/mL chymostatin, and 5 μg/mL pepstatin A (Sigma)]. We previously found that this lysis buffer solubilizes 100% of NKCC2 from TAL suspensions. The total protein content in each sample was measured in duplicate by colorimetric assay using Bradford’s method (Pierce Biotechnology). Equal amounts of protein (50–75 μg) were incubated overnight at 4°C with streptavidin-coated agarose beads (10%) in lysis buffer. Beads were pulled down by centrifugation, and the supernatant was reincubated with streptavidin-coated agarose beads (10%) for 2 h at 4°C. The supernatant was saved for the determination of intracellular NKCC2, whereas beads were centrifuged and pooled with the beads from the first round. Beads were then washed twice in lysis buffer, twice in high-salt buffer (500 mM NaCl, 50 mM HEPES, pH 7.4), and twice in no-salt buffer (50 mM HEPES, pH 7.4). Biotinylated proteins were extracted from the beads by boiling in 60 μL SDS-loading buffer containing 50 μM dl-dithiothreitol (DTT) and 5% β-mercaptoethanol. Proteins were resolved by SDS-PAGE (6% gels), and NKCC2 present in the membrane was detected by Western blotting.

### In Vivo Gene Silencing

First, we designed four siRNA sequences for ACTN4-targeted regions of the rat’s mRNAs. Then we synthesized nude siRNAs and tested their efficiency and selectivity by transfecting a rat kidney cell line (NRK-52E). Once silencing efficiency and specificity were confirmed by Western blot (Supplemental Fig. S1), we chose siRNA no. 3 (Supplemental Fig. S1, red box) to design shRNA and produce purified adenovirus particles. The target sequences for ACTN4 silencing were 21 nucleotides from the rat genes (5′-UCAACGAACUGGACUACUAUU-3′) which were introduced into adenovirus as described previously (28). Nude small interfering RNAs (siRNAs) were synthesized with the silencer siRNA construction kit from Applied Biosystems (Carlsbad, CA) and tested in vitro in NRK-52E cells from the American Type Culture Collection (Manassas, VA). Sense and antisense sequences spaced by a loop sequence ( TTCAAGAGA) were subcloned between the 5′ AflII and 3′ SpeI sites in the Adenovector-pMIGHTY (Viraquest, North Liberty, IA) to produce adenoviral particles coding short hairpin RNAs (shRNAs) for ACTN4. The constructs were sequenced, and replication-deficient adenoviral particles were assembled by Viraquest Inc. (Iowa City, IO), as we previously described (26).

The creation of ACTN4 guide RNAs was performed as previously described (29, 30). Briefly, we selected four guide RNAs targeting the exon 2 genomic region of mouse ACTN4 with high activity and minimum off-target by using Horizon CRISPR designing tool. We tested four gRNAs targeting ACTN4 in neuroblastoma Neuro 2a/HF-Cas9-ROSA26 cells and selected the most effective guide, gRNA no. 2 (Supplemental Fig. S2, red box), which decreased ACTN4 protein expression by 70%–80%, and we packed this gRNA into AAV (AVV-U6-gRNA-ACTN4). The sequence for the selected gRNA was 5′- AAGTGACCTGGCTGCACACC-3′ (PAM: AGG). We obtained the SpCas9-HF1 vector from Addgene (No. 138556) and subcloned it downstream of the NKCC2 promoter using the cloning site: HindIII-EcoRV into the pAD5 adenovirus packing vector. AV-expressing HF-Cas9 under control of the NKCC2 promoter (AV-pNKCC2-Cas9) was generated and purified by Viraquest Inc.

### In Vivo Adenovirus-/Adeno-Associated-Mediated Gene Transfer

Gene transfer to the renal medulla was performed as we previously described (12, 31, 32). Briefly, rats were anesthetized, shaved, and cleaned. The left kidney was exposed via flanked incision, fatty tissue removed, and both the renal artery and vein clamped for a maximum of 8 min. Adenovirus (1 × 10^12^ particles/ml) was loaded into PE50 tubing connected to a nanoliter syringe pump (Harvard Apparatus, Holliston, MA) set at 20 µl/min. A 30-gauge needle was attached to the other end of the tubing to inject the viruses into the renal outer medulla. Five injections (20 µl each) were made in 6 min (100 µl total volume), and the clamp was removed from the renal artery and vein after 8 min. After 7 days (for Ad-shRNA) or 14 days (for Ad-NKCC2-Cas9), the left kidney was removed and medullary TALs isolated as described earlier.

### Immunofluorescence Labeling

The kidney was fixed by retrograde perfusion through the abdominal aorta with freshly prepared 4% paraformaldehyde (PFA). Then kidneys were stored in 4% PFA for a day before embedding and proceeding with the tissue. Sections of the kidney were processed for H&E immunostaining. Colabeling of ACTN4 and NKCC2 was performed as described previously (14). Briefly, paraffin-embedded sections were first deparaffinized with xylene and then rehydrated gradually through 100%, 95%, and 70% ethanol to water. The sections were then unmasked by heat-induced antigen retrieval with citric acid buffer (10 mM, pH 6.0). After blocking nonspecific binding by 1% BSA, sections were incubated with primary antibody rabbit anti-NKCC2 (carboxyl terminus) 1:50 dilution (produced by GenScript for the Ortiz laboratory) at 4°C overnight. This was followed by 1:100 of Alexa-Fluor 488 donkey anti-rabbit IgG (Jackson ImmunoResearch, West Grove, PA) for 1 h at room temperature (RT). To label ACTN4, the sections were blocked again with 1% BSA and then incubated with 1:50 dilution of ACTN4 monoclonal antibody (clone D3; Santa Cruz, Dallas, TX). We have confirmed the specificity of this antibody by testing it in ACTN4 gRNA silencing sample from a rat’s kidney (Supplemental Fig. S5) at 4°C overnight. The epitope used to generate this antibody is -RCQKICDQWDNLGSLTHSRREALEKTEK. This was followed by incubating with 1:200 of Alexa-Fluor 647 goat anti-mouse IgM (Molecular Probes, Eugene, OR) for 1.5 h at RT. At the end, the sections were counter-stained with DAPI at 1:2,000 dilution for 5 min at RT. Slides were imaged with a Leica confocal microscope TCS SP8 multi-photon/confocal (×63, 1,024 × 1,024; Leica Microsystems, Germany) using spectral detectors set for maximal emission peak and minimal crossbleed.

### GST Pull-Down and Mass Spectrometry

A GST fusion protein comprising GST fused with full-length ACTN4 was obtained from Dr. Hung-Ying Kao (Department of Biochemistry, Case Western Reserve University). GST construct served as a control. The constructs were separately transformed by heat sock into competent *E. coli* and induced to express the construct by incubation with 0.18 mM IPTG at 28°C for 10 h. Bacterial proteins were extracted and incubated with glutathione-conjugated Sepharose beads (GE Healthcare) to purify the induced protein. TAL protein lysates (100 µg) were incubated with purified GST alone or GST-ACTN4 overnight at 4°C after precleaning the lysate with glutathione beads alone for 1 h. GST or GST-ACTN4 pull-downs were isolated using glutathione-agarose beads (ThermoFisher). The beads were extensively washed three times with low-salt (0.01 mM NaCl) and three times with high-salt (500 mM NaCl) buffer and then spun and submitted to the proteomics core of Michigan State University (East Lansing, MI) for LC-MS/MS identification of interacting proteins as we performed before (14). Identified protein interactions with ACTN4 were graphed with STRING (EMBL, string-db.org) high confidence (0.7) interactions.

### Immunoprecipitation

This was performed using a method described previously by our laboratory (14, 26). Briefly, freshly isolated medullary TALs were lysed as described above in materials and methods, *Steady-State Surface NKCC2 Levels*, and equal amounts of protein (100 μg) were incubated at 4°C with proteinA/G-coated agarose beads (50% slurry) in lysis buffer. Then, precleared protein lysates were incubated with 2 µg of NKCC2 antibody (carboxyl terminus), and the beads were washed the next day and subjected to Western blot.

### Western Blot

Proteins were eluted from streptavidin beads or TAL lysates by boiling (1 min) and then separated by centrifugation at 10,000 *g*, loaded into each lane of a 6% or 8% SDS polyacrylamide gel, separated by electrophoresis, and transferred to Immobilon-P polyvinylidene difluoride membranes (PVDF) (Millipore, Bedford, MA). Membranes were blocked and blotted as described (11). To detect actinin-4, we used mouse monoclonal actinin-4 (23) (gifted by Dr. Vivian Tang, University of Illinois), rabbit NKCC2 (carboxyl terminus), and mouse monoclonal GAPDH (Millipore, CA). HRP-labeled secondary antibodies were detected by chemiluminescence and quantified by densitometry (11, 13). Expose time and amount of protein loaded were optimized so that optical densities were linear.

### Urine and Ion Excretion in Metabolic Cage Studies

Metabolic cage urine and ion excretion were measured as we described previously (14). Briefly, rats were placed in metabolic cages and allowed to get adjusted to the cages for 4 days. For bumetanide response experiments, rats were trained to eat all their food between 9:00 AM and 6:00 PM, and food was restricted throughout the night. The next morning at 9:00 AM, rats were given 1 g of chocolate pudding, which they ate within 5 min. We did this for 4 days. On *days 4* and *5*, urine was collected for 14 h to record baseline urine volume and Na excretion. On *day 5*, 20 mg/kg bumetanide (as per the average weight of the rats) was mixed with the chocolate pudding, and urine was collected every 4 h to measure urine volume and urinary sodium content for 10 h. Volume was measured by weight, and Na was measured with an electrode (CareLyte; Diamond Diagnostics, Holliston, MA).

### Acute Blood Pressure Measurement

We performed acute blood pressure measurements in anesthetized rats as described before (33). Fourteen to sixteen days after viral transduction in the left kidney, rats were anesthetized with 2%–5% isoflurane (Piramal Pharma Limited, Telangana, India) and placed on a heating pad to maintain constant body temperature. A tracheotomy was performed using PE-260 tubing (Fisher Scientific, Chicago, IL) for spontaneous breathing of room air. Then the femoral artery was catheterized using PE-50 tubing to monitor femoral blood pressure (BP) directly using a Statham pressure transducer (Viggo-Spectramed, Oxnard, CA). The pressure transducer was connected to a Gould recorder (Valley View, OH) for recording BP as we did before (34). After a 30-min equilibration period, blood pressure was recorded for another 5 min. Blood pressure recordings for systolic blood pressure and mean blood pressure from each rat were exported to excel, and the first 2.5-min average readings were pooled for each group.

### Statistics

Results are expressed as means ± SE. One-way ANOVA was used to determine differences between means in treatments with only two groups. When there were more than two groups, we used two-way ANOVA with Tukey’s multiple comparisons test. For most comparisons between two groups, *P* < 0.05 was considered significant.

## RESULTS

### Expression of ACTN4 in the Thick Ascending Limb and Interaction with NKCC2 and Other Proteins

**Figure 1.**
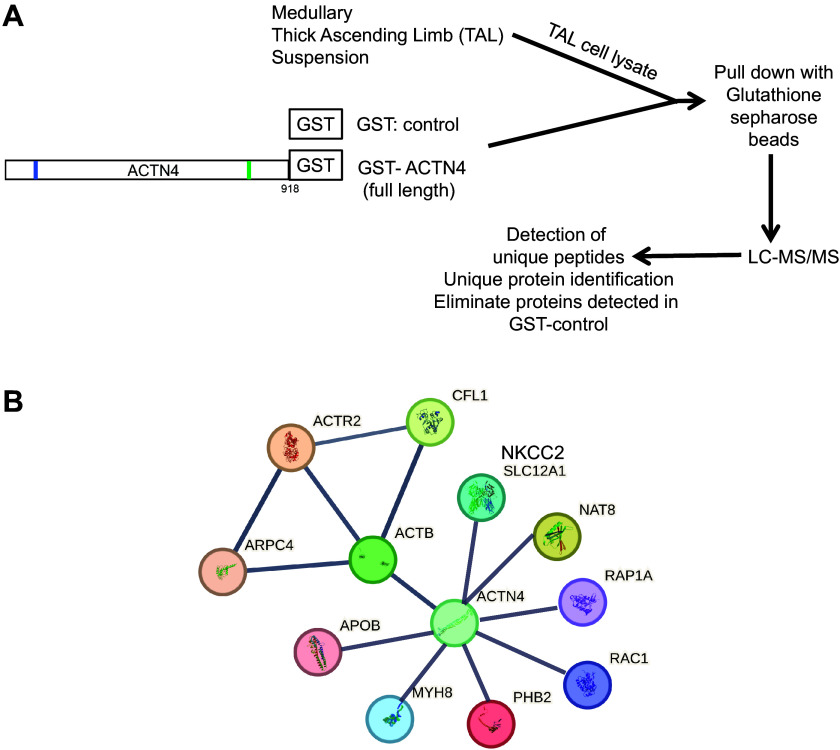
Identification of ACTN4 interacting proteins by LC-MS/MS. *A*: schematic diagram of the method used to identify ACTN4 interacting partners by LC-MS/MS in lysates from mTAL suspensions. *B*: recreated protein-protein interaction map by STRING software (EMBL) identified in GST pull-down assays with GST-ACTN4 (full-length) fusion proteins. ACTN4, actinin-4; GST, glutathione-*S*-transferase; mTAL, medullary thick ascending limb; NKCC2, Na^+^/K^+^/2Cl^−^ cotransporter.

**Figure 2.**
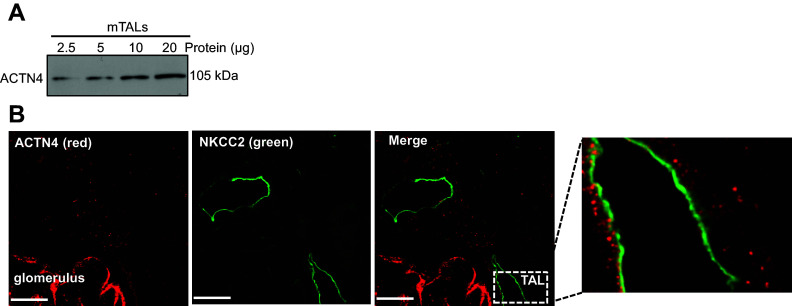
ACTN4 is coexpressed in TALs with NKCC2. *A*: representing immunoblot of ACTN4 indicating ACTN4 protein expression in rat mTAL corresponding to reported mass of 105 kDa (*n* = 3) (blot exposed time: 30 s). *B*: representative images showing immunofluorescence labeling of rat kidney cortex section for ACTN4 (red) and NKCC2 (green) in paraffin-embedded rat kidney sections, indicating expression and coexpression of ACTN4 and NKCC2 in the same TAL cells, in addition to prominent ACTN4 labeling in glomerular podocytes (*n* = 3). An additional image of glomerular labeling of ACTN4 is provided in Supplemental Material. Scale bar, 10 µm. ACTN4, actinin-4; TAL, thick ascending limb; NKCC2, Na^+^/K^+^/2Cl^−^ cotransporter.

Expression and function of ACTN4 in the podocytes have been widely studied (17). However, there are no studies of ACTN4 in the nephron or TALs. First, we used a proteomics screen to identify interacting proteins of ACTN4 in the TAL. We generated and purified glutathione-*S*-transferase-fusion ACTN4 (GST-full-length ACTN4) to pull down protein lysates of mTALs. We purified this protein from bacteria and used it to pull down the interacting proteins from lysates obtained from freshly isolated rat medullary TALs (Fig. 1*A*). A GST control pull-down was run simultaneously to identify nonspecific interactions with GST alone. As shown in Table 1 and Fig. 1*B*, NKCC2 and other trafficking proteins were identified only in GST-ACTN4 pull-downs [including actin, cytoplasmic 1 (ACTB), actin-related protein 2 (ACTR2), actin-related protein 2/3 complex subunit 4 (ARPC4), Annexin A1/A2 (ANXN1/2), cofilin-1 (CFL1), myosin-8 (MYH8), *N*-acetyltransferase 8 (NAT8), prohibitin-2 (PHB2), Ras-related protein Rap-1A (RAC1), Ras-related protein Rap-1A (RAP1A)]. Next, we confirmed the expression of ACTN4 in isolated rat medullary TALs. A single band at the expected molecular weight of ACTN4 (∼105 kDa) was observed in TAL lysates by Western blot (Fig. 2*A*). Then we tested whether ACTN4 is coexpressed in TALs in a similar location as NKCC2 by immunolabeling of ACTN4 and NKCC2 and confocal microscopy in kidney sections. We observed that, as expected, ACTN4 is abundantly expressed in glomerulus (Fig. 2*B* and Supplemental Fig. S6) but also in the nephron, including TALs labeled with NKCC2 in the subapical space, where ACTN4 labeling was punctate and vesicular. No labeling was detectable in the absence of a primary antibody (Supplemental Fig. S6).

**Table 1.** Summary of trafficking and endocytosis proteins identified by MS in the actinin-4 of TALs

Spot	Mr, kDa	No. of Peptides	Seq. Cov.	Protein ID
53	41,963.90	1	25.50	Cluster of actin, cytoplasmic 1 (ACTB)
54	44,734.70	1	3.05	Actin-related protein 2 (ACTR2)
55	19,667.40	1	11.30	Cluster of actin-related protein 2/3 complex subunit 4 (ARPC4)
71	38,831.00	1	13.00	Cluster of Annexin A1
72	38,680.20	1	4.13	Cluster of Annexin A2 (ANXN2)
73	536,031.10	3	0.21	Cluster of apolipoprotein B (APOB)
89	18,533.20	1	21.70	Cluster of cofilin-1 (CFL1)
170	42,851.70	2	2.04	Cluster of isocitrate dehydrogenase [NAD] subunit γ 1 (IDHG1)
204	166,640.50	7	5.09	Cluster of myosin-8 (MYH8)
205	24,809.10	5	5.88	Cluster of *N*-acetyltransferase 8 (NAT8)
216	52,562.30	6	2.38	NADH dehydrogenase iron-sulfur protein 2 (NDUS2)
238	33,313.60	2	20.50	Cluster of prohibitin-2 (PHB2)
255	21,441.30	4	3.69	Cluster of Ras-related C3 botulinum toxin substrate 1 (RAC1)
256	20,987.30	2	5.98	Cluster of Ras-related protein Rap-1A (RAP1A)
274	120,600.40	7	4.57	Cluster of solute carrier family 12 member 1 (SLC12A1)

Mr, molecular weight; Seq. Cov., sequence coverage; TALS, thick ascending limbs.

**Figure 3.**
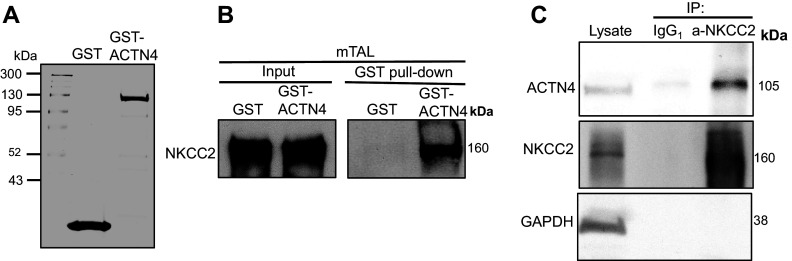
ACTN4 interacts with NKCC2 in TALs. *A*: Coomassie stain of bacterial recombinant purified GST-ACTN4 (full length). *B*: Western blot representing glutathione-GST pull-down of full-length ACTN4 and detection of NKCC2 by Western blot, which is absent in GST alone from TAL lysates (*n* = 3) (blot exposed time: 60 s). *C*: representative Western blot of immunoprecipitation (IP) of NKCC2 from TAL lysates and pull-down of ACTN4 (*n* = 3) (blot exposed time: 10 s). ACTN4, actinin-4; GST, glutathione-*S*-transferase; NKCC2, Na^+^/K^+^/2Cl^−^ cotransporter; TAL, thick ascending limb.

To confirm that ACTN4 interacts with NKCC2, we used purified GST-ACTN4 (35) and GST as a control (Fig. 3*A*). We observed that NKCC2 was detected in GST-ACTN4 pull-down but not in GST alone (Fig. 3*B*). To test whether endogenous ACTN4 interacts with NKCC2 in native TAL lysates, we performed coimmunoprecipitation in TAL lysates. We observed that ACTN4 coimmunoprecipitated with NKCC2 (Fig. 3*C*). Altogether, these results indicate that ACTN4 is expressed in the TALs and interacts with NKCC2.

### Silencing of ACTN4 in the Renal Medulla by shRNA Increases Surface NKCC2

**Figure 4.**
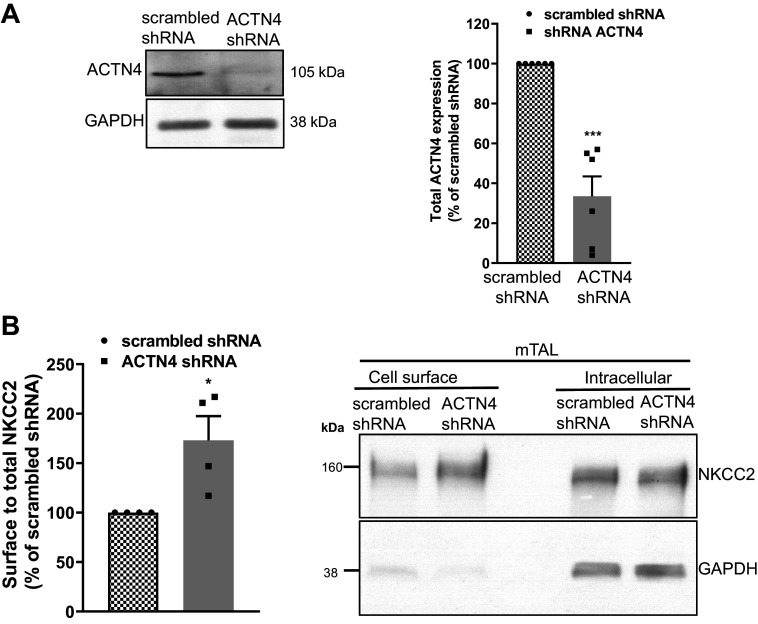
Silencing of ACTN4 by shRNA increases surface NKCC2 expression in TALs. *A*: representative Western blot showing decreased ACTN4 expression after 7 days after adenovirus-mediated gene silencing with Ad-shRNA-ACTN4 compared with control Ad-scrambled-shRNA in Sprague-Dawley rats (blot exposed time: 40 s). Quantification of ACTN4 after silencing in vivo in mTALs: ACTN4 expression was 33.5 ± 23.5% of the scrambled-shRNA. Error bars represent ±SE (*P < 0.01, ***P < 0.001, one-way ANOVA, *n* = 6) vs. scrambled-shRNA. *B*: quantification of steady-state surface NKCC2 after silencing ACTN4 in mTALs as a fraction of total NKCC2 in control (scrambled-shRNA) or Ad-shRNA-ACTN4. Steady-state surface NKCC2 expression was increased by 73 ± 24%. Error bars represent ±SE (*P < 0.02, one-way ANOVA, *n* = 4) (gray bar). A representative surface biotinylation experiment showing steady-state surface and intracellular NKCC2 in mTALs from rats transduced in vivo with ACTN4 or scrambled-shRNA vs. scrambled-shRNA (blot exposed time: 10 s). ACTN4, actinin-4; NKCC2, Na^+^/K^+^/2Cl^−^ cotransporter; TAL, thick ascending limb; mTALs, medullary thick ascending limbs.

To determine whether deletion of ACTN4 affects the functional role of NKCC2 in TALs, we silenced ACTN4 by using shRNA in the renal medulla. Then, we used adenoviruses to deliver shRNAs to the outer medulla in vivo as we did before (12) and determined the time course for maximal silencing in rat TALs. Six to seven days after injection, ACTN4 expression in medullary TAL suspensions was 61 ± 13% lower (*P* < 0.01, one-way ANOVA, *n* = 6) (Fig. 4*A*) compared with TALs from the control kidney injected with scrambled-shRNA adenovirus. We used these mTAL suspensions and surface biotinylation to measure surface NKCC2 and found that the surface-to-total NKCC2 ratio was increased by 73 ± 16% in TALs from rats transduced with ACTN4 shRNA (*P* < 0.05, one-way ANOVA, *n* = 4). GAPDH was only detected in the intracellular but not in surface fraction (Fig. 4*B*). Importantly, ACTN4 silencing did not affect total NKCC2 expression.

### CRISPR/Cas9-Mediated Gene Silencing of ACTN4 in TALs Increases Surface NKCC2

**Figure 5.**
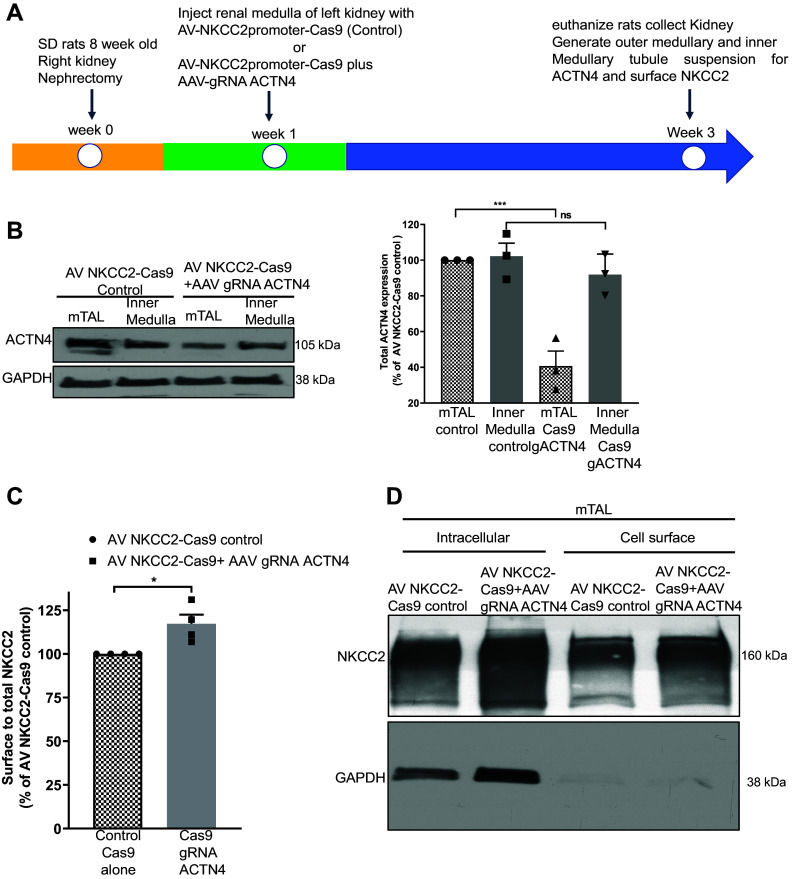
Silencing of ACTN4 in TALs by CRIRPR/Cas9 increased NKCC2 surface expression. *A*: scheme for timeline used for direct injection of AV-NKCC2promoter-Cas9 or AV-NKCC2promoter-Cas9 + AAV-ACTN4-gRNA. *B*: representative blot and quantification showing ACTN4 protein expression in mTALs after direct injection of AV-NKCC2-Cas9-control or AV-NKCC2-Cas9 + AAV-ACTN4-gRNA in inner and outer medullary tubule suspensions from normal Sprague-Dawley rats. After silencing of ACTN4 with targeted gRNA, ACTN4 expression was decreased by 59 ± 14%. Error bars represent ±SE (*P < 0.01, ***P < 0.001, two-way ANOVA, Tukey’s multiple comparison test, *n* = 3) vs. NKCC2-Cas9-control (blot exposed time: 30 s). *C*: quantification of steady-state surface NKCC2 after silencing ACTN4 in mTALs as a fraction of total NKCC2 in TALs isolated from control AV-NKCC2-Cas9-control or AV-NKCC2-Cas9 + AAV-ACTN4-gRNA and steady-state surface NKCC2 expression was increased by 20 ± 5%. Error bars represent ±SE (*P < 0.02, one-way ANOVA, *n* = 4) vs. NKCC2-Cas9-control. *D*: representative blot showing surface NKCC2 as a fraction of total NKCC2 in TALs isolated from control AV-NKCC2-Cas9-control or AV-NKCC2-Cas9 + AAV-ACTN4-gRNA in the mTAL (blot exposed time: 30 s). ACTN4, actinin-4; mTALs, medullary thick ascending limbs; NKCC2, Na^+^/K^+^/2Cl^−^ cotransporter; SD, Sprague-Dawley; TALs, thick ascending limbs.

Adenovirus-mediated shRNA induces a decrease in ACTN4 expression in all cells transduced with adenoviruses and is not specific to TAL cells. To improve our system and make it selective to TALs, we used a second approach in which we used the CRISPR/Cas9 system to knock down ACTN4. To silence ACTN4 and then measure TAL-mediated ion transport in rats, we removed the right kidney (unilateral right uninephrectomy) and then allowed the rats to recover for a week. Then, we cotransduced the renal medulla of the left kidney with both AV-pNKCC2-Cas9 (50 µL) and AAV-ACTN4 gRNA (50 µL) (Fig. 5*A*). Fourteen days after transduction, we isolated TALs from the outer medulla and inner medullary tubules from the inner medulla and found that ACTN4 expression was decreased by 59 ± 10% in TALs but not significantly changed in the inner medulla (which does not have NKCC2) compared with control rats injected only with Cas9 (*P* < 0.01, two-way ANOVA, Tukey’s multiple comparison test, *n* = 3) (Fig. 5*B*). These data support the specificity of the Cas9 approach to decrease ACTN4 in medullary TALs in vivo. In a separate group of rats, 2 wk after transduction of the left kidney with AV-NKCC2-Cas9 + AAV-gRNA ACTN4, we next studied if ACTN4 silencing in TALs would enhance surface NKCC2. We measured steady-state surface NKCC2 expression in TALs suspensions. We found that the surface-to-total NKCC2 ratio was increased by 20 ± 6% in TALs from rats transduced with Cas9-ACTN4 (*P* < 0.05, *n* = 4) (Fig. 5*C*). The intracellular protein GAPDH was not detected at the surface fraction in surface biotinylation experiments (Fig. 5*D*). In addition, ACTN4 silencing did not affect total NKCC2 expression. These observations indicate that ACTN4 mediates steady-state surface NKCC2 expression.

### Silencing of ACTN4 Enhances TAL-Mediated NaCl Reabsorption

**Figure 6.**
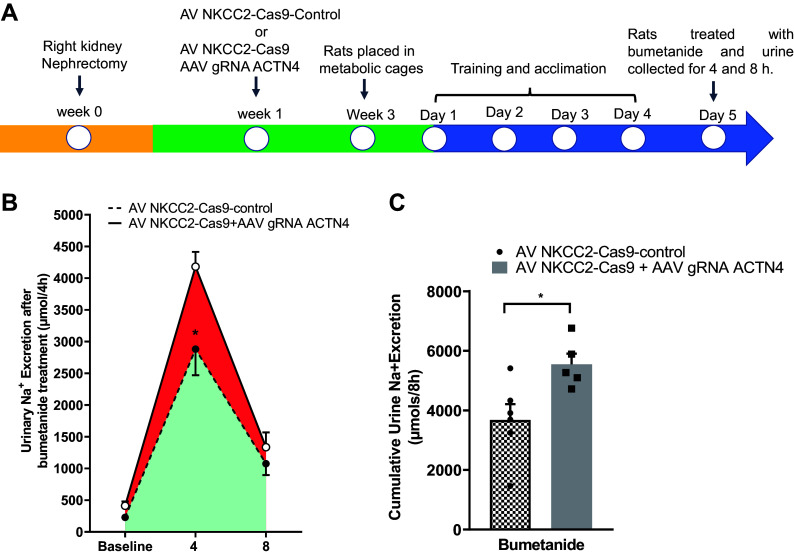
In vivo knockdown of ACTN4 in TALs with Cas9 enhances bumetanide-induced natriuresis. *A*: timeline for protocol including single nephrectomy, direct injection of AV-NKCC2promoter-Cas9-Control or AV-NKCC2-Cas9 + AAV-ACTN4-gRNA and metabolic cages to collect the urine. *B*: urinary Na^+^ excretion after bumetanide treatment was significantly higher in rats transduced with NKCC2promoter-Cas9 + AAV-ACTN4-gRNA compared with rats transduced with of AV-NKCC2promoter-Cas9 alone. Error bars represent ±SE (*P < 0.05, two-way ANOVA, *n* = 5) vs. NKCC2-Cas9-control. *C*: cumulative urinary sodium excretion after bumetanide-induced UNa excretion was higher in rats transduced with AV-NKCC2-Cas9 + AAV-ACTN4-gRNA compared with rats transduced by AV-NKCC2-Cas9-control. Error bars represent ±SE (*P < 0.02, one-way ANOVA, *n* = 5) vs. NKCC2-Cas9-control. ACTN4, actinin-4; NKCC2, Na^+^/K^+^/2Cl^−^ cotransporter; TALs, thick ascending limbs.

To test whether deletion of ACTN4 in TALs enhances NKCC2-mediated NaCl reabsorption in TAL, we measured NKCC2-mediated NaCl transport in vivo. In a separate group of uninephrectomized rats, we transduced the left kidneys with AV-NKCC2-Cas9 + AAV-gRNA-ACTN4 or AV-NKCC2-Cas9 alone (control). After 3 wk, rats were placed in metabolic cages and we collected urine (Fig. 6*A*). At baseline, 24-h collection showed that urine volume or Na was not significantly different between groups. To estimate NKCC2-mediated NaCl absorption in vivo, we measured the acute diuretic and natriuretic effects of the NKCC2 inhibitor bumetanide. We found that bumetanide-induced diuresis and natriuresis was higher in rats transduced with AAV-gRNA-ACTN4, compared with rats transduced with AV-NKCC2promoter-Cas9 alone (Cas9 control: 2,880 ± 381 vs. Cas9-ACTN4: 4,181 ± 190 µmol Na/4 h, *n* = 5, *P* < 0.05) (Fig. 6*B*). We found that the cumulative urine excretion over 8 h after bumetanide administration was significantly higher in rats with silenced ACTN4 (AV-NKCC2-Cas9: 3,841 ± 709 vs. AAV-gRNA-ACTN4: 5,546 ± 622 µmol Na/8 h, *P* < 0.05, *n* = 5) (Fig. 6*C*). We next studied if silencing of ACTN4 in TALs affects the blood pressure on these rats. At the end of the experiment, rats were anesthetized and blood pressure was measured to study any differences. We found that baseline systolic blood pressure was increased by 26 ± 06% mmHg in rats transduced with Cas9-ACTN4 (AV-NKCC2-Cas9: 79 ± 07 mmHg vs. AAV-gRNA-ACTN4: 105 ± 13, *P* < 0.05, *n* = 4 each group; Supplemental Fig. S3). We did not find any significant difference in body weight of rats transduced with Cas9-ACTN4 vs. control (*P* < 0.97, *n* = 5) (Supplemental Fig. S4). These data indicate that silencing of ACTN4 in TALs enhances TAL-mediated NaCl reabsorption indicative of higher NKCC2-mediated NaCl transport.

## DISCUSSION

In this study, we report that ACTN4 is expressed in TALs and interacts with NKCC2. Our data indicate that ACTN4 mediates NKCC2 trafficking from the apical surface of TALs and that decreasing ACTN4 expression enhanced surface NKCC2 levels and TAL-NaCl reabsorption. Our data suggest that ACTN4 could be a negative regulator of NKCC2-mediated NaCl reabsorption involved in NKCC2 endocytosis from the apical surface of TALs.

The first data suggesting that ACTN4 could regulate NKCC2 came from our previous studies, in which we found that ACTN4 pulled down with recombinant ALMS1 in TAL lysates (14, 36). Here, we extended those studies to find that full-length ACTN4 pulled down NKCC2 and a number of other trafficking and signaling proteins. We confirmed the ACTN4-NKCC2 interaction by coimmunoprecipitation of NKCC2 from TAL lysates. Thus, our data suggest that ACTN4 might be part of a complex that regulates NKCC2 expression at the apical surface in TALs. Despite the importance of ACTN4 in structural support in podocytes and cell mobility in cancer, the specificity and role of ACTN4 in nephron NaCl transport were previously unrecognized. Decreasing ACTN4 expression with shRNA in the outer medulla or via Cas9 in TALs enhanced surface NKCC2 levels without affecting total NKCC2 expression. It is not clear which mechanism increases surface expression, but it most likely involves a decrease in the rate of NKCC2 endocytosis. We previously found that NKCC2 undergoes constitutive endocytosis, and inhibiting this process enhances surface NKCC2. Moreover, ACTN4 interacts with ALMS1, which we showed is involved in NKCC2 endocytosis (14). α-actinins (ACTNs) (36) are essential cytoskeletal proteins that cross-link actin filaments (37), and ACTN4 has been involved in endocytosis of NHE3 in fibroblasts (38). Here, we found that ACTN4 also pulled down Annexins1/2, which are involved in Ca-dependent membrane trafficking; ARPC4, an actin-binding protein enhanced by vasopressin in collecting ducts (39); cofilin-1, involved in actin depolymerization and endocytosis; and RAC1. Based on our previous findings, we speculate that ACTN4 could mediate NKCC2 endocytosis and potentially could play a role as an inhibitor of NKCC2 activity by decreasing NKCC2 at the apical surface of TALs. Under baseline conditions, silencing of ACTN4 in TALs increased systolic blood pressure, suggesting that the ACTN4-NKCC2 pathway could have a direct effect on blood pressure regulation. However, this remains a hypothesis, and future experiments need to further address this in conscious animals under conditions of low- or high-salt diets. Future studies will explore the dynamic nature of these interactions and whether they control the rate of NKCC2 endocytosis.

To our knowledge, the functional role of ACTN4 in TALs has not been documented yet. However, in humans, gain-of-function mutations in ACTN4 cause FSGS in podocytes (40). In contrast, mice deficient in ACTN4 do not have congenital nephrosis, suggesting that alterations in ACTN4 lead to podocyte damage and the development of FSGS by causing subtle cytoskeletal changes (16). However, the role of ACTN4 in nephron ion transport has not been studied. Single nucleotide polymorphisms in ACTN4 are associated with hypertension in GWAS (41), suggesting it may regulate renal NaCl reabsorption or affect blood pressure by regulating overall renal function. We found that decreasing ACTN4 expression in TALs increased TAL-NaCl absorption and surface NKCC2 expression. We used two systems to decrease ACTN4 expression in the renal medulla of SD rats: *1*) adenovirus-mediated shRNA and *2*) CRISPR/Cas9-mediated decrease of ACTN4 in TALs. Both maneuvers decreased ACTN4 expression in TALs by 50%–60%, in line with what we reported before for other genes using AV-mediated shRNA. We elected the Cas9 system to measure NaCl reabsorption because it decreases ACTN4 in TALs driven by the NKCC2 promoter Cas9, but not in other nephron segments. The decrease in ACTN4 was sufficient to increase surface NKCC2 by 25% and also enhanced bumetanide-induced natriuresis by 35%. Using shRNA or CRISPR/Cas9 reduced ACTN4 expression by ∼60%. Yet the stimulatory effect on surface NKCC2 was not as marked with CRISPR/Cas9. The reason for this difference is unclear. It is possible that decreasing ACTN4 expression in cells surrounding TALs (as it occurs with shRNA) has a further inhibitory signaling effect, independently from the direct NKCC2-ACTN4 interaction. Nevertheless, both approaches significantly increase surface NKCC2 and TAL-mediated NaCl reabsorption. Overall, our data provide an evidence that ACTN4 does play a role in regulating renal NaCl reabsorption, in part by interacting with NKCC2, controlling its surface expression, and affecting TAL-mediated NaCl absorption. It is not clear how ACTN4 expression and trafficking are regulated in renal tubules. Renal ACTN4 expression is decreased in glomeruli by a high-fat diet in mice (42), something that may impact tubular NaCl reabsorption. In addition, ACTN4 expression was also evident in other nephron segments, but its role in NaCl transport in other segments remains to be studied. Overall, there is so little known about ACTN4 outside the glomerular podocytes that multiple questions remain about the mechanism of action, regulation of signaling pathways, and regulation of ACTN4 expression in the nephron and TAL specifically.

We previously demonstrated that trafficking of NKCC2 to the apical membrane is important for the regulation of NaCl absorption by the TALs. Thus, proteins that regulate NKCC2 surface expression are likely to affect NaCl transport by the TAL. Here, we demonstrate that ACTN4 interacts with NKCC2 (and other proteins), and that is, gene silencing increases surface NKCC2 and TAL-mediated NaCl reabsorption. These are the first studies demonstrating a role for ACTN4 in the distal nephron NaCl reabsorption and underscore the need for additional studies on this protein, known to be involved in glomerular disease.

## DATA AVAILABILITY

Data will be made available upon reasonable request.

## SUPPLEMENTAL MATERIAL

Supplemental Fig. S1: https://doi.org/10.6084/m9.figshare.27080191.

Supplemental Fig. S2: https://doi.org/10.6084/m9.figshare.27085660.

Supplemental Fig. S3: https://doi.org/10.6084/m9.figshare.27085708.

Supplemental Fig. S4: https://doi.org/10.6084/m9.figshare.27085723.

Supplemental Fig. S5: https://doi.org/10.6084/m9.figshare.27085735.

Supplemental Fig. S6: https://doi.org/10.6084/m9.figshare.27085744.

## GRANTS

This work was supported by the National Institute of Health-NIDDK Grant R01DK131114A1 (to P. A. Ortiz) and Henry Ford Fund.

## DISCLOSURES

No conflicts of interest, financial or otherwise, are declared by the authors.

## AUTHOR CONTRIBUTIONS

D.M. and P.A.O. conceived and designed research; D.M., T.-D.L., D.L.P., and P.A.O. performed experiments; D.M., T.-D.L., and P.A.O. analyzed data; D.M. and P.A.O. interpreted results of experiments; D.M. prepared figures; D.M. drafted manuscript; D.M. and P.A.O. edited and revised manuscript; D.M., T.-D.L., and P.A.O. approved final version of manuscript.
